# Direct current electrical fields inhibit cancer cell motility in microchannel confinements

**DOI:** 10.1038/s41598-025-87737-7

**Published:** 2025-02-07

**Authors:** Benjamin Karem Naggay, Saeed Khomeijani Farahani, Xu Gao, Andrew Holle, Ralf Kemkemer

**Affiliations:** 1 Department of Life Sciences, Reutlingen University, 72762 Reutlingen, Germany; 2 Reutlingen Research Institute, Reutlingen University, 72762 Reutlingen, Germany; 3 Charité Campus Benjamin Franklin, Charité-Universitätsmedizin Berlin, 12203 Berlin, Germany; 4 Department of Biomedical Engineering, National University of Singapore, Singapore, 117411 Singapore; 5 Department of Cellular Biophysics, Max Planck Institute for Medical Research, 69120 Heidelberg, Germany

**Keywords:** Cellular motility, Cell invasion, Extracellular matrix, Metastasis

## Abstract

The capability of cells to sense and respond to endogenous electrical fields plays a crucial role in processes like nerve regeneration, wound healing, and development. In vitro, many cell types respond to electrical fields by migrating along the corresponding electrical field vectors. This process is known as galvano- or electrotaxis. Here we report on the combined impact of micro-confinements and direct current electrical fields (dcEFs) on the motility of MDA-MB-231 human breast cancer cells using a self-developed, easy-to-use platform with microchannels ranging from 3 $\upmu$m to 11 $\upmu$m in width and 11 $\upmu$m height. We found that MDA-MB-231 cells respond to exogenous electrical fields ranging from 100 mV mm$^{-1}$ to 1000 mV mm$^{-1}$ with altered cell motility depending on the confinement size. Our data show an overall inhibited galvanotaxis in confinements, while in contrast an enhancing effect in unconfined galvanotaxis is found. The application of direct current electrical fields to microchannels not only caused a reduction in migration speed but also decreased the number of permeating cells. By applying 1000 mV mm$^{-1}$, single-cell permeation could be prevented in confinements of 5 $\upmu$m and smaller.

## Introduction

The invasion of cells into surrounding tissue, their dispersion through the blood- and lymphatic system, and the formation of metastases transform a locally growing tumor into a systemic and life-threatening disease with a poor prognosis^1^. In the metastatic event, the active migration of tumor cells plays an essential role. This complex process depends on numerous cell-intrinsic factors and the chemical and physical properties of the extracellular surrounding^2,3^. While the influence of extrinsic chemokines or accumulated genetic mutations on metastasis development has been widely studied, a better understanding of physical cues and their interplay with biochemical changes in the tumor environment is still necessary^1,3–5^. Research on the interplay of cells and physical signals from their environment, may provide significant insights into metastatic mechanisms and serve as the basis for new noninvasive therapeutic treatments^6^. Therefore, some research has been performed to enlighten the dependency of dynamics of tumor cell motility on physical cues for a better understanding of cancer metastasis^7–13^. In particular, the impact of endogenous (in vivo) occurring electrical fields (EFs) on various processes of tumor development has been addressed in several studies but details on mechanisms are still to be revealed^2,14–16^. The first documentation of (in vivo) measured electrical currents occurred in 1843 by du Bois-Reymond^17^. He discovered small electrical currents in the region of a small cut on his finger. Further discoveries revealed that these small currents (referred to as dcEFs) occur in various physiological and pathological processes like embryonic development, cancer metastasis, or wound healing^18–21^. Electrical fields in vivo can range from a few millivolts up to several hundred, with typically experimentally measured values of up to 200 mV mm$^{-1}$ in strength^19^. Zhu et al. demonstrated, for example, that intra-tumoral electrical potentials in 4T1 breast tumor allografts can range from 30–465 mV mm$^{-1}$^22^. These magnitudes of electrical fields pose a quite substantial cue for cells, as the order in which cells can sense EFs is only approximately in the order of one magnitude compared to a four-orders-of-magnitude in chemotaxis^23^. Applying dcEFs of similar sizes (5–1000 mV mm$^{-1}$) in cell culture experiments can alter cell division, polarity, shape, and motility^7,8,14,24^. Furthermore, many cell types, like tumor cells^10,25^, epithelial cells^26^, mesenchymal cells^24^, stem cells^27^, or immune cells^28^, respond with a directed migration towards the cathode or anode, called galvano- or electrotaxis^29,30^. It has been observed in various cell types that the directionality of the migration is typically increasing with increasing field strength, however, the origin of the directionality is poorly understood. One of the more widely suggested mechanisms underlying this phenomenon is the asymmetrical redistribution of epidermal growth factors or membrane lipid receptors^31,32^. Moreover, intracellular signaling pathways like the RTK-PI3K or ERK1/2 pathway are found to play a role in electrotaxis^31,33^. In addition to electrical signals, cell motility is often impaired by confinements. For example, during the tumor extravasation process, cells migrate either through the extracellular matrix (ECM), along channels between bundled collagen fibers, or squeeze between other cells during wound healing^3,34,35^. One limitation of 2D experiments is the integration of these geometric confinements. Therefore, such constraints are often simulated with microchannels as they can be manufactured with versatile but precise dimensions specific to the scientific question. As previously reported, physical confinements can alter the migratory phenotype of tumor cells from a mesenchymal to an amoeboid migration phenotype^36–39^. In a study conducted by Balzar et al., the so-called mesenchymal to amoeboid transition (MAT) for MDA-MB-231 cells started to occur for confinements smaller than 20 $\upmu$m and showed a dominant mesenchymal migration till confinements reached 6 $\upmu$m. In confinements smaller than 3 $\upmu$m the only active migration mechanism is the amoeboid migration, where the necessary actin polymerization for mesenchymal migration has not been observed^36^.

**Figure 1:**
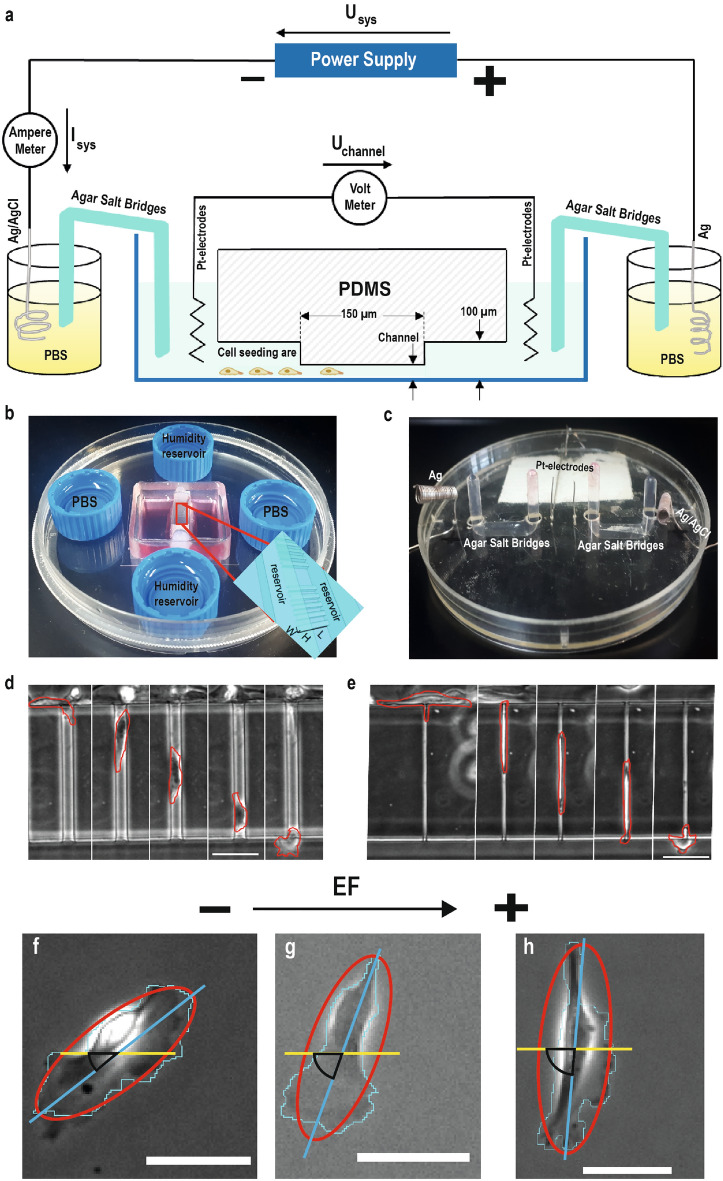
Experimental set-up for measuring cell motility in response to dcEF in microchannel confinements. (**a**) Schematic representation of the chip platform (side view). Each set-up contains one coiled Ag/AgCl (cathode) and Ag (anode) electrode placed into PBS reservoirs connected with agar salt bridges to the cell migration chamber filled with cell culture media. (**b**) Image of the bottom part of the used set-up, containing the migration chamber with channels and electrolyte reservoirs. (**c**) Image of the top part (lid) of the used set-up, containing two agar-salt bridges, two Pt-wires for the voltage measurement, one Ag/AgCl electrode (right), and one Ag electrode (left). (**d**) Images of a time-lapse video with an MDA-MB-231 cell permeating an 11 $\upmu$m channel. For better visualization, the cell was encircled manually. (**e**) Images of a time-lapse video with an MDA-MB-231 cell permeating a 3 $\upmu$m channel. For better visualization, the cell was encircled manually. (**f**–**h**) Exemplary images of a cell reorienting in a 500 mV mm$^{-1}$ electrical field. The yellow line resembles the orientation of the electrical field vectors. The red ellipse resembles the ellipse used for fitting and gaining the orientation angle (black marking). The blue line resembles the major axis of the cells taken in the electrical fields. (**f**) Cell at the time point t0. (**g**) Cell at the time point t12. (**h**) Cell at the time point t24. Scale bar 50 $\upmu$m.

**Figure 2:**
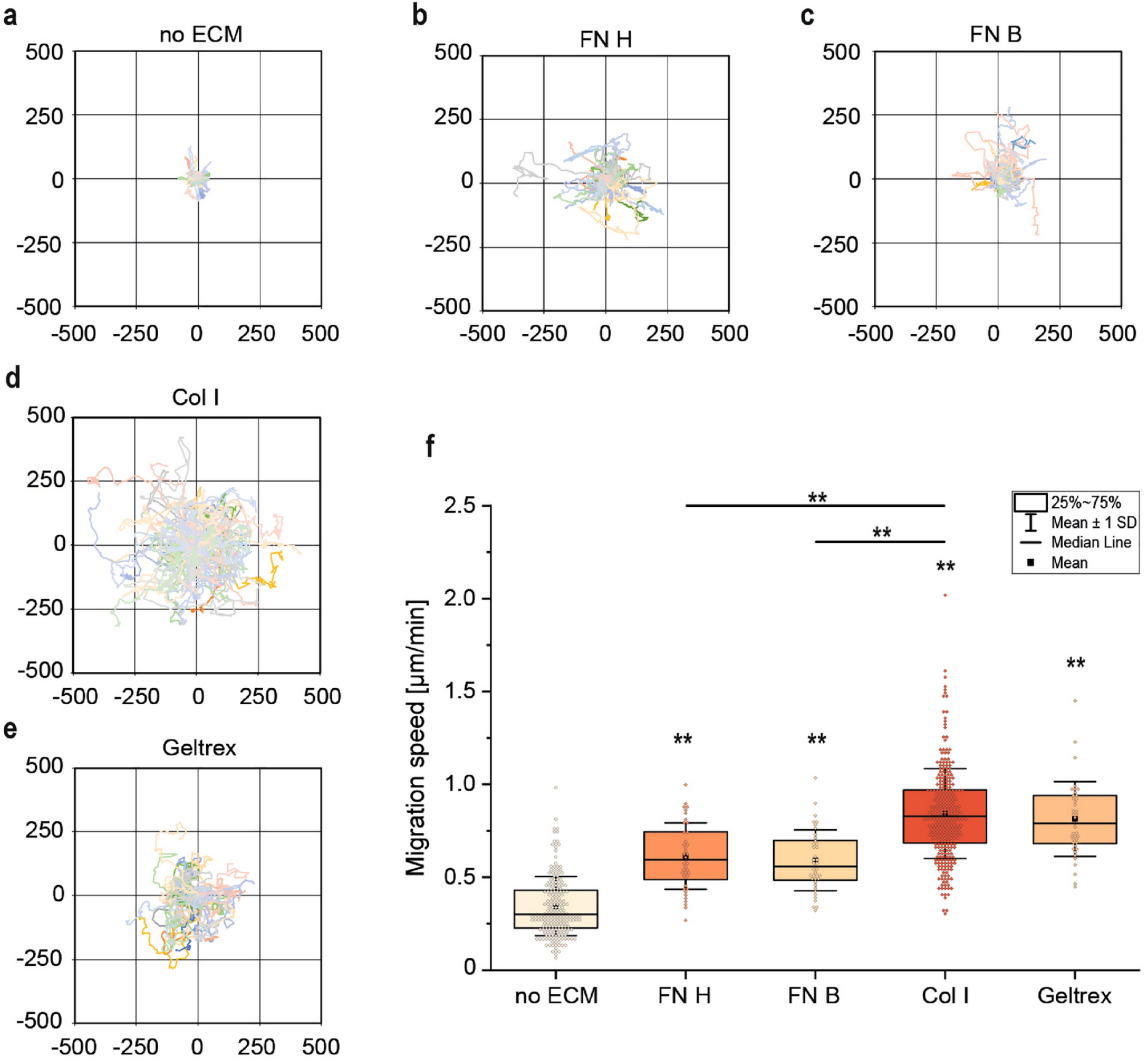
(**a**) Migration tracks of MDA-MB-231 cells on a glass substrate without coating (n = 63). (**b**) Migration tracks of MDA-MB-231 cells on a glass substrate coated with FN H (n = 40). (**c**) Migration tracks of MDA-MB-231 cells on a glass substrate coated with FN B (n = 40). (**d**) Migration tracks of MDA-MB-231 cells on a glass substrate coated with Col I (n = 90). (**e**) Migration tracks of MDA-MB-231 cells on a glass substrate coated with $\hbox {Geltrex}^{\textrm{TM}}$ (n = 40). (**f**) Migration speed of individual cells in unconfined conditions in dependency of extracellular protein coating (n_cells_ $\ge$ 40). (** p < 10$^{-6}$ in comparison to control with no ECM, Mann-Whitney-test).

To investigate the behavior of tumor cells in micro-confinements while being exposed to dcEFs in and above the physiological range, we systematically examined the migration of MDA-MB-231 cells in a microfluidic cell migration set-up. This set-up has been developed and built, allowing the application of electric fields across two reservoirs connected by an array of microchannels (Fig. 1a–c). We performed galvanotaxis experiments with channel widths ranging from 3  to 11 $\upmu$m and electrical field strengths from 100 to 1000 mV mm$^{-1}$ and observed the migration of the MDA-MB-231 epithelial cancer cells by microscopy (Fig. 1d, e and Supplementary Vid. V1 and V2). We could demonstrate a strong dependency of tumor cell motility on the degree of confinement and electrical field strength. By confining the MDA-MB-231 cells, we caused an increase in migration speed but when applying direct current electrical fields, the cells reacted with a decreased speed. Furthermore, we could show that it is possible to prevent tumor cells from migrating through presented microchannels, by exposing them to EFs above the physiological range. In addition, we could replicate the two distinct motion behaviors of MDA-MB-231 cells according to the degree of confinement.

## Results

### Migration and galvanotaxis in unconfined conditions

To analyze the influence of different adhesive coatings on the motility of MDA-MB-231 cells, we used three matrix proteins, namely collagen type I (Col I), human fibronectin (FN H), and bovine fibronectin (FN B), and one basement membrane extract ($\hbox {Geltrex}^{\textrm{TM}}$) to modify the galvanotaxis chamber by simple physisorption. We found that all coating materials caused a significant (p = 6.1E−16 and smaller) increase in median migration speed (ṽ) compared to the untreated glass substrate (Fig. 2). Col I coating caused the highest increase in migration speed (ṽ = 0.83 $\upmu$m min$^{-1}$) by nearly tripling the median speed compared to the no ECM condition (ṽ = 0.30 $\upmu$m min$^{-1}$). $\hbox {Geltrex}^{\textrm{TM}}$ (ṽ = 0.79 $\upmu$m min$^{-1}$) had a comparable effect on migration speed as collagen type I. Human (ṽ = 0.59 $\upmu$m min$^{-1}$) and bovine (ṽ = 0.56 $\upmu$m min$^{-1}$) fibronectin coating also increased migration speed similarly by almost doubling the displayed speed of the cells compared to the control condition (Fig. 2). As Col I is an integral part of many ECMs and enabled the highest migration speed in our experiments, Col I was used as the sole coating protein in all galvanotaxis experiments.

**Figure 3:**
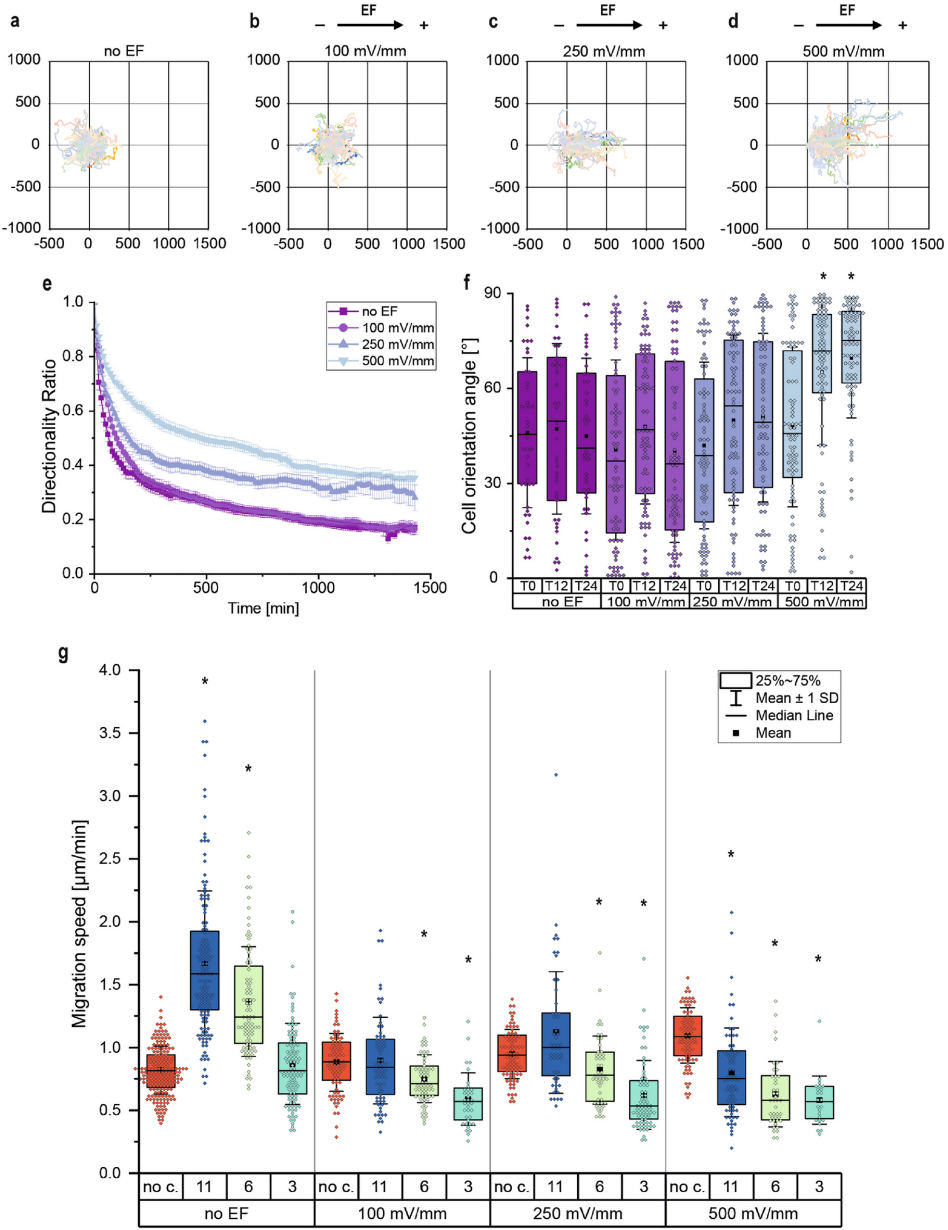
Cell motility of MDA-MB-231 cells on Col I coating is differently altered by electrical fields in confined compared to unconfined conditions (no c.) when monitored over 24 h. (**a–d**) Migration trajectories of tracked cells with 0:0 set as the origin for all cells (n_cells _$\ge$ 80). (**e**) Quantification of the directionality ratio in unconfined conditions (n_cells_ $\ge$ 80). (**f**) Quantification of cell orientation angle in unconfined conditions after 0 h of EF application (t0), after 12 h of EF application (t12), and after 24 h of EF application (t24). 0$^{\circ }$ equals orientation along the electrical field vectors and 90$^{\circ }$ to a vertical orientation (n_cells_ $\ge$ 41). (**g**) Quantification of migration speed in $\upmu$m min$^{-1}$ (n_cells_ $\ge$ 24). (* p < 0.05 in comparison to the unconfined condition with corresponding field strength, Mann-Whitney-test).

**Figure 4:**
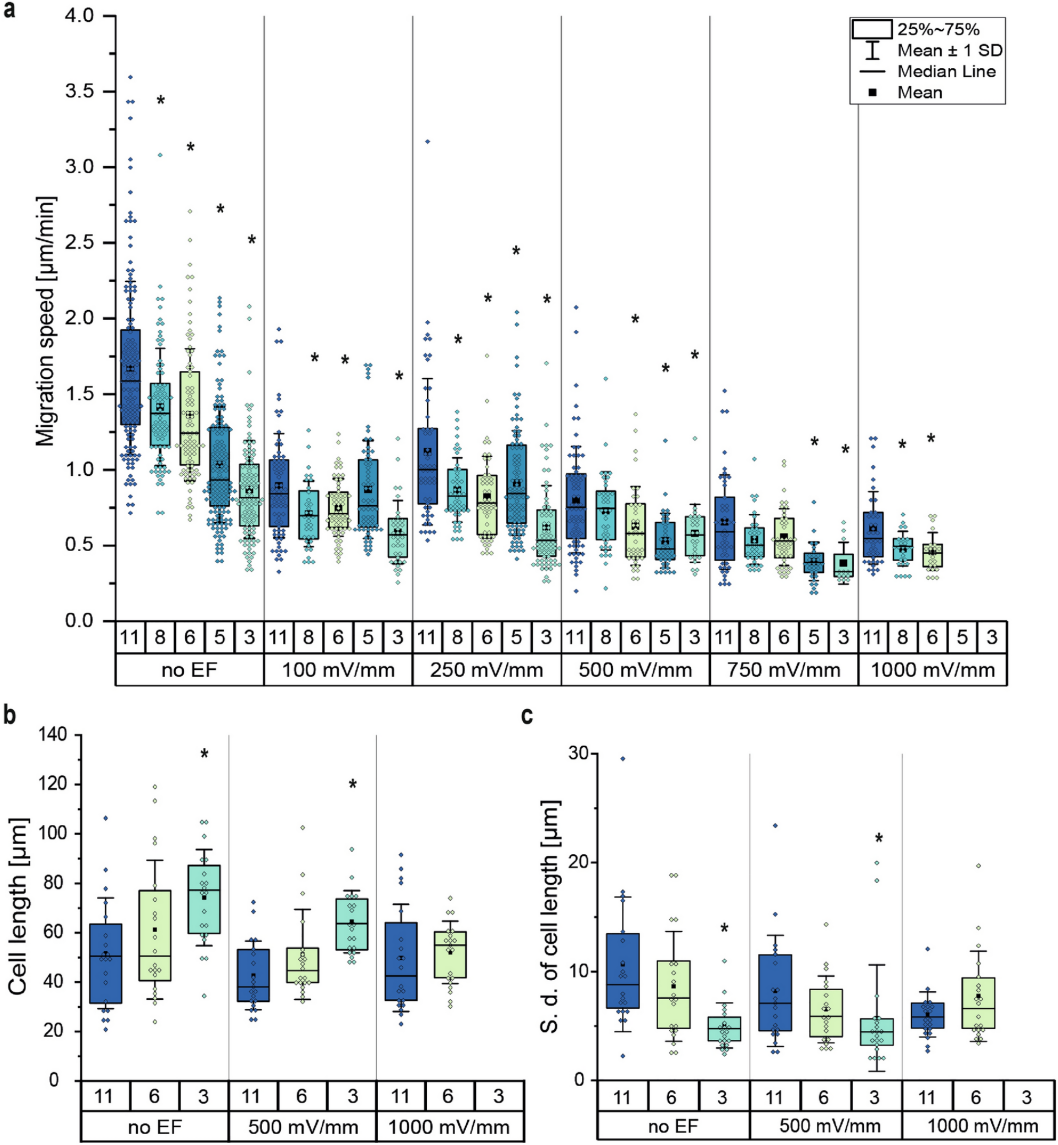
MDA-MB-231 cells sense and respond to different confinements and electrical field strengths. (**a**) Migrations speed in dependency of channel size and EF strength (n_cells_ $\ge$ 12 for each condition). (**b**) Distribution of average cell length (mean value of the single cell) of permeating cells tracked inside the respective microchannels (n_cells_ = 20). (**c**) Distribution of the standard deviation for mean cell length of single cells permeating the channels over time (n_cells_ = 20). (* p < 0.05 in comparison to 11 $\upmu$m-confinement with according field strength, Mann-Whitney-test).

**Figure 5:**
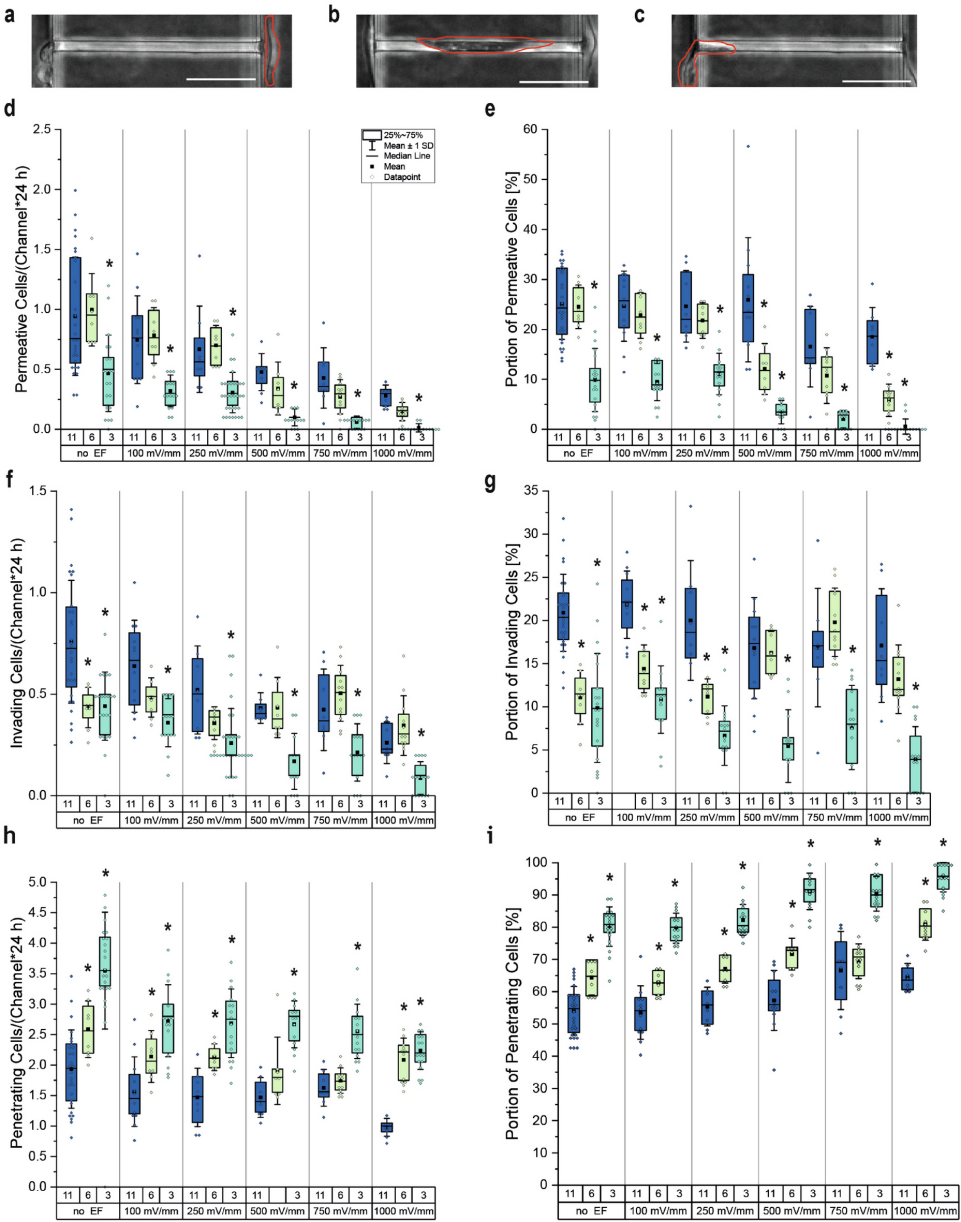
Channel interaction of MDA-MB-231 breast cancer cells. (**a**) Example of a permeating cell entering the channel on the cathode side and leaving it on the anode side. (**b**) Example of an invading cell entering the channel space fully but not leaving the channel on the anode side. (**c**) Example of a penetrating cell entering at least 15 μm into the channel space. Scale bar 50 $\upmu$m. (**d**) Quantification of permeating cells per channel over one day (24 h) at different electrical fields. (**e**) Proportion of permeating cells in different channel sizes at different electrical fields. (**f**) Quantification of invading cells per channel over one day (24 h) at different electrical fields. (**g**) Proportion of permeating cells in different channel sizes at different EFs. (**h**) Quantification of penetrating cells per channel over one day (24 h) at different electrical fields. (**i**) Proportion of permeating cells in different channel sizes at different electrical fields. (n_image sequence_ $\ge$ 8; * p < 0.05 in comparison to 11 $\upmu$m-confinement in according field strength, Mann-Whitney-test).

To investigate the impact of direct current electrical fields on cell motility and cell orientation without micro-confinements, we performed galvanotaxis experiments with three different field strengths within the physiological range, from 100 to 500 mV mm$^{-1}$ (Fig. 3a–g). Here the cell orientation angle is defined by fitting an ellipse on the analyzed cell, giving the major axis of the cell which is then set in relation to the electrical field vector (Fig. 1f–h). Applying dcEFs of 100 mV mm$^{-1}$ resulted in a significant (p = 0.018) increase in migration speed but had no effects on the directionality of the migration or the average cell orientation angle (Fig. 3b, e, f, and g). By applying electrical fields of 250 mV mm^-1^, tumor cells were notably affected in terms of migration speed and directionality but not in orientation when compared to the control without an applied EF (Fig. 3c, e, f, g). Comparing the impact of EFs with 100 mV mm$^{-1}$ to EFs with 250 mV mm$^{-1}$, only the directionality was substantially increased but no alteration of the migration speed was found. Applying an electrical field of 500 mV mm$^{-1}$, the tumor cells responded with a further increase in migration speed and directionality, and a significant (p = 2.8E−4 after 12 h of EF application (t12) and p = 5.7E−8 after 24 h of EF application (t24)) change in cell orientation to an orthogonal direction of the electrical field (Fig. 3d–g). Comparing migration speed, directionality, and orientation angle of the 500 mV mm$^{-1}$ experiments with the effect of 250 mV mm$^{-1}$ on cell motility all investigated parameters were significantly increased (p = 1.8E−5 and smaller).

### Migration and galvanotaxis in confinements

To determine the combined influence of confinements and electrical fields on MDA-MB-231 cell migration, we tracked only cells migrating one at a time and not dividing in the channels with three different sizes while being exposed to electrical fields over 24 h. Here we define three different confinements namely, slight confinements (11 $\upmu$m channel width), medium confinements (6 $\upmu$m channel width), and strong confinements (3 $\upmu$m channel width). We found that by confining tumor cells slightly, migration speed was significantly increased (p = 1.4E−62) compared to unconfined conditions (no c.) but this effect was lost when confinements got more severe (Fig. 3g). In contrast to unconfined migration, applying electrical fields to cells migrating through the different microchannels had an inhibitory effect on their cell motility. Applying 100 mV mm$^{-1}$ and 250 mV mm$^{-1}$ dcEFs in experiments with 11 $\upmu$m confinements resulted in migration speeds comparable to those in unconfined conditions, while 500 mV mm$^{-1}$ provoked a considerably reduced migration speed (ṽ_no c._ = 1.09 $\upmu$m min$^{-1}$ compared to ṽ_11_ = 0.75 $\upmu$m min$^{-1}$). In 3 $\upmu$m and 6 $\upmu$m confinements, applying EFs resulted in a substantially lower speed for all field strengths when compared to cells with unconfined migration. By plotting the position of the leading edge of the cells in the channels over time (kymographs), we could also observe a change in directionality bias (Supplementary Fig. 1). With an increase in electrical field strength, the migration of the MDA-MB-231 cells was found to be more persistent than with no applied electrical fields.

To investigate the progression of the migration speed in dependence on the different degrees of confinement and electrical fields above the physiological range (EFs > 500 mV mm$^{-1}$) we additionally analyzed 5 $\upmu$m and 8 $\upmu$m confinements as well as 750 mV mm$^{-1}$ and 1000 mV mm$^{-1}$ electrical fields (Fig. 4a). When applying an EFs of 750 mV mm$^{-1}$, MDA-MB-231 cells showed similar migration speeds for 6 $\mu$m (ṽ = 0.53 $\upmu$m min$^{-1}$) and 11 $\upmu$m (ṽ = 0.59 $\upmu$m min$^{-1}$) channels, and only for 3 $\upmu$m (ṽ = 0.33 $\upmu$m min$^{-1}$) a significantly slower (p = 0.001 compared to 11 $\upmu$m) migration speed was measured. By increasing the field strength to 1,000 mV mm$^{-1}$, cells showed a significantly lower (p = 0.074) migration speed in medium compared to slight confinements. Strikingly, by applying the same electrical fields to stronger confinements, we were able to prevent single cells from migrating through the presented channels. Furthermore, our data shows that different electrical fields in the physiological range (100 mV mm$^{-1}$ < EFs < 500 mV mm$^{-1}$) applied to 3 $\mu$m confinements did not affect migration speed differently. For all field strengths, except at 500 mV mm$^{-1}$, cells in 8 $\mu$m channels showed similar migration speeds as in 6 $\upmu$m channels. At this field strength, cells showed a significantly (p = 0.028) higher migration speed in 8 $\mu$m channels compared to 6 $\mu$m ones. In contrast to the other channel sizes, applying EFs of 100 mV mm$^{-1}$ in 5 $\upmu$m channels (ṽ = 0.76 $\upmu$m min$^{-1}$) did not affect migration speed equally, leading to a similar migration speed as cells showed in experiments with 11 $\upmu$m channels (ṽ = 0.84 $\upmu$m min$^{-1}$). However, when applying high dcEFs between 500 mV mm$^{-1}$ and 1000 mV mm$^{-1}$, cells in 5 $\upmu$m channels showed similar migration speeds as cells in 3 $\upmu$m channels and also stopped migrating through the channels for electrical fields with 1000 mV mm$^{-1}$.

To assess the motility phenotype within channels, we separately tracked the leading edges and the rear of permeating cells. We could observe that cells exhibited two distinct motion behaviors: either the so-called “push-and-pull” migration pattern, which is characterized by changing the cell length frequently, or a “sliding behavior”, which is characterized by a uniform movement of the cell’s front and rear^40^. These migration behaviors were quantified by plotting the average cell length and the standard deviation (S. D.) of the length of individual cells while migrating through the channels (Fig. 4b, c and Supplementary Fig. 2). We could observe that cells inside slight confinements show the shortest cell length with l̃ = 50.54 $\upmu$m, while cells in strong confinements show the longest cell length with l̃ = 77.25 $\upmu$m (Fig. 4b). However, cell length showed no noteworthy dependency on the applied electrical fields. Comparing the median length of cells in 6 $\mu$m confinements (l̃ = 50.59 $\upmu$m) to the length of cells in 11 $\upmu$m channels (l̃ = 50.54 $\upmu$m) for no EF conditions no difference was found, nor when applying different EFs. Only cells in 3 $\mu$m confinements showed a significant increase (p = 0.0016 for no EF and p = 2.9E−5 for 500 mV mm$^{-1}$) in median cell length compared to 11 $\upmu$m confinements independent of the applied electrical field. While the variation in cell length occupied by cells inside the channels shows an increasing progression with declining channel widths, the standard deviation of the cell length decreases with declining channel widths (Fig. 4c). Cells in medium confinements showed no notable different impact in S. D. compared to slight confinements at all electrical field conditions. Cells in strong confinement were found to be significantly smaller compared to 6 $\upmu$m (p = 0.013) and 11 $\upmu$m (p = 2.9E−5) channels without an applied EF but were found to only be significantly different for 11 $\upmu$m (p = 0.02) channels when applied 500 mV mm$^{-1}$. By comparing the standard deviation of the cell length for the individual channel size at the different EF strengths, for 3 $\upmu$m and 6 $\upmu$m channels no dependency between both parameters could be found. Only in 11 $\upmu$m channels at 1000 mV mm$^{-1}$, a significant change in S. D. (p = 8.4E−4) compared to the untreated cells could be found. Showing that the “push-and-pull” migration pattern is changed to a “sliding behavior” when manipulating cells with EFs.

### Microchannel interaction

To investigate the invasive potential in dependence on electrical fields and confinements, we classified the interaction of the cells with the microchannels by using three categories as described by Rolli et al.^40^. The first category is “*permeative cells*”, for cells that migrate fully through the channels, entering them on one side (cathode-side) and leaving them on the opposite (Fig. 5a). The second category is defined as “*invasive cells*”, counting cells that enter the channels fully (Fig. 5b), however, later stop migrating onward by staying inside the channel or reversing their migration direction backward and leaving the channel at the point of their entrance. The third category is “*penetrating cells*”, for cells that penetrate a minimum of 15 $\mu$m into the presented channels but do not enter the channel fully (Fig. 5c).

By plotting the distribution of the number of cells permeating through one channel over 24 h, we found a strong dependency between the channel width and the number of cells permeating (Fig. 5d, Supplementary Fig. 3a). This dependency was altered by the application of electrical fields as 100 mV mm$^{-1}$ and 250 mV mm$^{-1}$ showed an inhibitory effect for all channel dimensions, except for 5 $\upmu$m ones. When applying EFs of 500 mV mm$^{-1}$ and above, a drastic decrease in cell number could be observed in all channels, and almost completely preventing cells from permeating through 3 $\upmu$m and 5 $\upmu$m channels at 1000 mV mm$^{-1}$. When plotting the portions of permeating cells, we could see similar trends in the change of the percentage (Fig. 5e, Supplementary Fig. 3b). Noticeably the percentage of cells permeating through 8 $\upmu$m and 11 $\upmu$m channels showed no dependency on electrical field strengths in the physiological range (100–500 mV mm$^{-1}$). Plotting the distribution of the absolute number of cells invading a channel over one day also showed a strong dependency on the degree of confinement (Fig. 5f, Supplementary Fig. 3c). Here all dimensions from 6 $\upmu$m and below allowed a similar number of cells to invade the channel space, while the application of electrical fields caused in general a decline in the number of invading cells with stronger electrical fields. Converting these numbers in portions of cells interacting with the channels, no clear dependency between channel width, EF strength, and the portion of invading cells could be observed (Fig. 5g, Supplementary Fig. 3d). When displaying the distribution of the number of cells penetrating one channel over 24 h, no dependency between the degree of confinement and the number of cells could be found (Fig. 5h, Supplementary Fig. 3e). By exposing cells to electrical fields also no dependency between the number of penetrating cells and the EF strength could be found. In contrast to the lack of dependency between the number of penetrating cells and the channel dimensions, the portion of penetrating cells shows an increase in percentage with the decline in channel width (Fig. 5i, Supplementary Fig. 3f). This percentage increase was reinforced by the application of electrical fields.

Summarized, our data shows that by confining cells in 3 $\upmu$m or 5 $\upmu$m channels, the probability of cells entering and successfully migrating through the channels is significantly reduced compared to 11 $\upmu$m and 8 $\upmu$m channels. In 6 $\upmu$m confinements, MDA-MB-231 cells were as likely to migrate through the presented channels as in 11 $\upmu$m ones but were significantly unlikely to remain in the channels. Applying dcEFs to the confinements amplified the inhibitory effect of the confinements on the invasiveness, reducing the percentage of permeating cells with the increase of EF strength. Furthermore, not only the likelihood of a successful permeation was decreased by the application of the electrical fields but also the number of cells permeating was lowered.

## Discussion

Extra- and intravasation of cells play a key role in cancer progression. Understanding the influence of exogenous or endogenous electrical fields on this process might offer new strategies for targeting tumor metastasis, as galvanotaxis of cancer cells could play an important role in metastasis events, especially considering that natural direct current electric fields have been detected in various biological contexts, including within tumors^14,18,19^. Our goal was to investigate the combined influence of confinements and dcEFs on cancer cell motility to give further insights into the combined role of these two factors in the metastatic process of cancer cells. As previously reported by other groups, we could also show that MDA-MB-231 cells detect and respond to a wide range of electrical field strengths with a directed migration towards the anode^8,13,15,25,41,42^. In healthy breast epithelium, the typical transepithelial electrical potential (TEP) is approximately +30 mV^43^. The direction of this electric potential (from negative to positive) with the anode placed in the lumen, is consistent with the initial path of breast cancer cell metastasis, where the cells migrate into the lumen, as noted by Faupel et al. in 1997^43^, and is consistent with the initial direction of metastasis of breast cancer cells as described by Wellings and Jensen in 1973^44^.

Our study confirmed previous reports on electrical fields affecting the motility of MDA-MB-231 cells in unconfined conditions. Similar to the Reports of Wu et al., Kim et al., or Pu et al. our cells responded to dcEFs with a directed and accelerated migration towards the anode^25,41,42^. While our cells reacted with a similar increase in migration speed at the same electrical field strengths as reported in these three papers, a notable change in the directionality occurred with electrical fields of 250 mV mm$^{-1}$ and higher, while Wu et al. reported a notable change at 150 mV mm$^{-1}$, and Pu et al. at 100 mV mm^-1^. These differences might be attributed to the different cell media and coating materials for Wu et al. and to the different media with a lower amount of serum used in the paper of Pu et al.^41,42^. In addition to previous papers, we analyzed the influence on the alignment of cells (orientation of the length axis) and could note a considerable change at 500 mV mm$^{-1}$ but not for smaller EFs. Biological cells aligning perpendicular to electric fields in in-vitro experiments is a well-documented phenomenon, particularly for elongated cells such as fibroblasts and epithelial cells^24,45,46^. We hypothesize that cells respond to strong direct current EFs by a change in their orientation along the electrical field vectors to reduce the stress experienced by the EFs. This reorientation reduces the cell volume orthogonal to the electrical field vectors and therefore reduces the electrical field the cells are exposed to.

Furthermore, we could show that confinements alone influence cell motility drastically. Exposing MDA-MB-231 cells to 11 $\upmu$m confinements boosts migration speed compared to unconfined conditions, but with decreasing channel dimensions, the initial effect gets lost (Fig. 3g and supplement Vid. V1 and V2). These findings fall in line with previously published data which show a similar impact on the migration speed of MDA-MB-231 cells in confinements close to 3 $\upmu$m^37,47–50^. For the impact of microchannels close to 11 $\mu$m, these mentioned papers report on different observations. While Holle et al. and Todorovski et al. report on a higher migration speed in small confinements than in wider channels, Afthinos et al., Mak et al., Irima et al., and Stöberl et al. report on a higher migration speed in wider channels than in small confinements^51^.In general, the dependency of the progression of cell migration speed with changes in the degree of confinement is no heterogeneous phenomenon, and is highly dependent on cell type, substrate stiffness, the density of ECM proteins, surface roughness and others^39,52–54^. One additional factor influencing the migration speed inside the different confinements might be attributed to the deformation of cell organelles^55,56^. Fluorescent imaging of MDA-MB-231 cells inside the confinements revealed that the cell nucleus in 5 $\upmu$m confinements was roughly half the width of the cell nucleus inside 11 $\upmu$m confinements. In contrast, in 3 $\upmu$m confinements, the nucleus showed approximately a third of the diameter compared to a nucleus 11 $\upmu$m wide channels (Supplementary Fig. 4). When comparing the length of the nucleus inside the confinements an elongation in 3 $\upmu$m and 5 $\upmu$m confinements could be observed compared to 11 $\mu$m confinements. These observations align with reports from Yixuan et al. where they report on similar deformation of the cell nucleus of MDA-MB-231 cells in confined environments^57^. This deformation of the nucleus and lateral forces to the channel walls might impede the fast migration through small channels.

The observation that applying 1000 mV mm$^{-1}$ to 11 $\upmu$m confinements changes the “push-and-pull” migration phenotype to a “sliding behavior” is reported for the first time. Based on the previous reports of Balzar et al. and Holle et al. mesenchymal migration is characterized by a high variation in cell length, while cells with an amoeboid migration show a low variation in cell length^36,37^. When assigning these phenotypes to our results, the mesenchymal migration in 11 $\upmu$m confinements may changes to an amoeboid migration with 1000 mV mm$^{-1}$.

While applying EFs to cells in unconfined conditions showed an enhancing effect on tumor cell migration, applying the same EFs had an inhibiting effect on the migration of the tumor cells in confinements. Whereas the application of 500 mV mm$^{-1}$ and higher always had a similar decreasing effect on migration speed for 3 $\upmu$m and 5 $\upmu$m (amoeboid-based), the same EFs show different decreasing effects depending on the limitation of mesenchymal migration. These observations and findings lead to the assumption that strong electrical fields may impact mesenchymal-based motility differently than amoeboid-based. Furthermore, we could show for the first time that the combinatory effects of confinement and electrical fields prevent cells from permeating through channels. This effect was only present in channels inducing a dominant amoeboid migration phenotype. Additionally, to the inhibitory effect on the migration speed, confinements in combination with electrical fields caused a reduction in the total number of cells interacting with the presented microchannels. With higher field strength a lower total number of cells interacted with the presented channels and were less likely to succeed in the process of permeation. This reduced number of cells permeating the channels might be attributed to the posed hypothesis on why cells reorient their cell in electrical fields to reduce the experienced EF.

Though a lot of research has been conducted in recent years, a complete mechanistic understanding of how cells respond to electric fields and the specific cellular pathways involved in sensing EFs remains elusive. EFs present a highly intricate stimulus, easily modulated in strength and direction, yet challenging to predict in its impact on cellular mechanisms and the surrounding microenvironment. It is widely assumed that EFs not only modify cellular behavior by affecting ion channels, ion distribution (such as asymmetric calcium ion flux and intracellular calcium), or the redistribution of membrane proteins but also influence charged elements within the microenvironment, including proteins and ions^7,14,18,19,21,26,58^. These effects pose challenges for a complete understanding, and the primary molecular mechanisms underlying these phenomena await full elucidation. On the other hand, these suggestions support the finding that galvanotaxis is a general and rather robust phenomenon across many cell types including cancer cells.

## Conclusion

For the first time, we report on the combinatory effects of direct current electrical fields and microconfinements on cell motility of MDA-MB-231 cells. Our presented data confirms, that applying direct current electrical fields up to 500 mV mm$^{-1}$ have no inhibitory influence on MDA-MB-231 motility in unconfined conditions, but presents a potent stimulus to control cell migration in terms of directionality, speed, or orientation angle of individual cells. By introducing MDA-MB-231 cells to confinements we could show that the migration speed in the presented microchannels was increased initially but this effect was lost the more narrow the channels became. Additionally, the stimulatory effects of EFs on cell motility in unconfined conditions are not present in confined conditions mimicking the migration through a 3D environment. Meaning under all confined conditions, in terms of electrical field strength and channel size, the application of an electrical field caused a decrease in migration speed compared to no EF conditions. Furthermore, when confined and exposed to dcEFs cells permeated less frequently through the presented channels, even stopping for EFs of 1000 mV mm$^{-1}$ when applied to confinements of 5 $\upmu$m and smaller. These observations highlight the different impacts of electrical fields depending on the degree of confinement and that already small electrical fields pose a potent stimulus for manipulating cells. Hence, this study might contribute to a better understanding of how electrical fields and pores in the ECM play a role in the metastatic progression of cancer. In addition, we show that EFs three to four times larger than physiologically occurring fields can alter and inhibit the migration of tumor cells, which may be a hint for the therapeutic use of EF.

## Methods

### MDA-MB-231 cell culture

For the experiments, the breast cancer cell line MDA-MB-231 (gifted from the Max Plank Society in Stuttgart, Germany) was used. The cells were cultivated in DMEM (ThermoFisher Scientific) supplemented with 10 % fetal calf serum (ThermoFisher Scientific), and 1 % penicillin/streptomycin (ThermoFisher Scientific). The cells were cultured inside an incubator at 37 $^\circ \textrm{C}$, and 5 % CO_2_ and passaged at a confluence of 80 %.

### Microfluidic platform design and fabrication

The channel designs were fabricated via standard photolithography techniques. Briefly, a two-step photolithography process for the fabrication of the silicon master was applied. The layouts of the structures were drawn with AutoCAD (Autodesk, USA), and used to fabricate the structures with a tabletop maskless aligner ($\upmu$MLA; Heidelberg-Instrument, Germany). The first, thin layer of SU-8 10 photoresist (MicroChem, USA) was spin-coated onto the freshly cleaned silicon wafer. After soft baking, the first structure was produced via exposure with the $\upmu$MLA using “Write Mode I”. Subsequently, the first structure was developed (mr-Dev 600; Microresist, Germany) and a second, much thicker layer, of SU-8 2075 photoresist (MicroChem, USA) was spin-coated on the wafer. The wafer was aligned to the structures from the first fabrication step and exposed with the “Write Mode III” to form the second structure and again developed with the mr-Dev 600 developer. For replica molding, the PDMS prepolymer (Sylgard 184; Dow Corning, Germany), was mixed in a ratio of 10:1. The mixed PDMS was cast over the structured silicon wafer placed in a petri dish and cured for 4 h at 80 $^\circ \textrm{C}$ in an oven. To seal the PDMS replica onto a glass slide, the glass slides were washed with acetone and then activated together with the PDMS replica for 30 s under an oxygen plasma with a pressure of 0.4 mbar and a power of 150 W. Gently pressing the PDMS on the glass allowed for permanent and irreversible bonding of the PDMS replica to the glass slide.

### Fabrication of Ag/AgCl electrodes

The electrical field was applied via one Ag/AgCl (cathode) and one Ag (anode) electrode, placed on opposite sides of the microfluidic platform. The electrodes were produced by electrochemical deposition of chloride ions on coiled silver wires 1 mm in diameter and 13 cm in length (TICAR Versand, Germany). The silver wires were coiled to increase the surface area and then placed in 1 M HCl (Fisher Chemical, Germany) together with platinum wires. The silver wires were chloritized for 5 min with 5 V, then removed and rinsed with deionized water.

### Fabrication of agar salt bridges

For the agar salt bridges customized glass tubes were filled with a 2-weight % agarose in PBS. For this, the agar solution was heated until boiling, then transferred into the glass tubes, and allowed to solidify for 5 min.

### Galvanotaxis experiments

Confined galvanotaxis experiments were performed in a customized cell migration platform (Fig. 1a–c), utilizing standard microfabrication techniques. Galvanotaxis experiments for unconfined conditions were performed with $\mu$-Slides (ibidi, Germany). Before cell-seeding the platforms were coated with either collagen type I, Geltrex^™^, human or bovine Fibronectin. Collagen type I (EDM Millipore Corp, Germany) was used with a concentration of 100 $\upmu$g mL$^{-1}$ and stored at 4 $^\circ \textrm{C}$ overnight. Geltrex^™^ (ThermoFisher Scientific, Germany) was used with a concentration of 5 mg cm$^{-2}$ and both fibronectin with 100 $\upmu$g mL$^{-1}$ and incubated for 30 min at 37 $^\circ \textrm{C}$. Afterward, the coating solution was replaced with PBS and then placed under ultraviolet (UV) light for 20 min. For the microchannel experiments, 5000 MDA-MB-231 cells were seeded on the cathode side of the chamber and allowed to adhere for 2 h before performing experiments. For the unconfined experiments, the cells were seeded inside the channels and also allowed to adhere for 2 h before performing experiments. For each condition, at least two individual experiments were performed for 24 h. Directionality ratio data are presented as mean ± SEM. Presented data in a boxplot diagram is presented with ± 25 % confidence intervals and ± SD. Statistical analysis was performed with Origin 2022b (OriginLab, Northampton, MA, USA) by using the Mann-Whitney test.

### Immunofluorescence staining and imaging

Cells seeded in microfluidic chips were fixed using 3.7 % paraformaldehyde at 37 $^\circ \textrm{C}$ for 45 min on a shaker. Following fixation, samples were rinsed with PBS and permeabilized with 0.2 % Triton X-100 for 15 min at RT. After three washes in PBS, cells were blocked with 1 % bovine serum albumin (BSA) dissolved in PBS containing 0.1 % Tween-20 for 30 min at RT. CF568 Phalloidin (1:100, Biotium, 00044) was applied for actin filament staining, and nuclei were counterstained with Hoechst 33342 (1:5000, Invitrogen, H3570) for 10 minutes at RT, followed by three PBS washes. Stained samples were imaged immediately using an Olympus FV3000 confocal microscope equipped with a 60X oil immersion objective (Olympus UPLXAPO 60X).

### Plasmid production and lentiviral transduction

NES-GFP-expressing (Nuclear export signal) MDA-MB-231 cells were generated through lentiviral transduction using the $\hbox {Lenti-X}^{\textrm{TM}}$ Packaging System (Takara Bio) in the presence of 5 $\mu$g mL$^{-1}$ polybrene (Sigma-Aldrich). The NES-GFP construct was delivered using a pCDH lentiviral plasmid. Fluorescently labeled cells were selected and enriched using a Sony SH800S Cell Sorter.

### Application and measurement of electrical values

The necessary electrical current set for the desired electrical field was calculated by Ohm’s law in the following form:

                                               $$I_{\textrm{set}}~=~E~*~\sigma ~*~A$$

where: E is the desired field strength (in mV mm$^{-1}$); $\sigma$ is the conductivity of the media (in mS mm$^{-1}$; 1.477 mS mm$^{-1}$ for all included studies); A is the summed cross-sectional area of the 80 individual microchannels or the cross-sectional area of the used ibidi chamber (in mm^2^); and I_set_ (I_sys_ in Fig. 1a) is the set current measured by the multimeter (in $\mu$A) which is adjusted in the unconfined experiments to match the electrical field strength in the confined experiments^9^. Electrical values (voltage (U_channel_) and electrical current) were measured simultaneously using two VC850 (Voltcraft) and the respective software. Values were taken every 10 s and later converted to an average value over 1 h.

### Microscopy and image analysis

Time-lapse phase-contrast images were collected using an inverted microscope (Zeiss Axiovert 200M; Zeiss, Germany) with a 10x objective (CP-Achromat; Zeiss, Germany). A customized live-cell chamber for the microscope was used to maintain cell culture conditions at 37 $^\circ \textrm{C}$, 5 % CO_2_, and relative humidity of 30 %. Time-lapse images were stacked and edited with ImageJ (Version 1.51; NIH, USA) and later used with the tracking software CellTracker (Version 1.1)^59^. The migration speed (instantaneous speed) was generated by using the software CellTracker which uses the accumulated migration vectors and the time needed to travel this distance to calculate the values^59^. Cells were tracked beginning with entering a channel until reaching the end with the cell front. For this paper only cells migrating one at a time and not dividing in the channels were selected for analysis. To generate migration tracks of the unconfined breast cancer cells the X and Y coordinates obtained from CellTracker were transferred into an Excel file and processed with macros to give the migration tracks of each cell based on the same starting point (X = Y = 0)^60^. The directionality ratio or meandering index gives the ratio between the straight-line length from the start- to the endpoint of the migration trajectory, divided by the length of the actual migrated distance^60^. The orientation angle was defined as the angle between the major axis of the ellipse fitted to the cell outline (using ImageJ) and the electric field vector (Fig. 1f–h).

### Statistical analysis

Directionality ratio, direction autocorrelation, and mean square displacement data are presented as mean ± SEM. Presented data in a boxplot diagram is presented with ± 25 % confidence intervals and ± SD. Statistical analysis was performed with Origin 2022b (OriginLab, Northampton, MA, USA) by using the Mann-Whitney-Test. Generated data is derived from at least two independent experiments and analyzed as individual cells if not stated otherwise.

## Supplementary Information

Supplementary Video 1.

Supplementary Figures.

Supplementary Video 2.

## Data Availability

Generated or analyzed data from this study are included in this published article. Raw data, images as well as voltage and current measurements are available upon request.
