# Supplementary material for: Direct current electrical fields inhibit cancer cell motility in microchannel confinements
Source: Sci Rep. 2025 Feb 7;15:4605. doi: 10.1038/s41598-025-87737-7 (PMC11806051; doi:10.1038/s41598-025-87737-7)
Supplement: Supplementary file 2 — Supplementary Figures. [file 41598_2025_87737_MOESM2_ESM.docx]

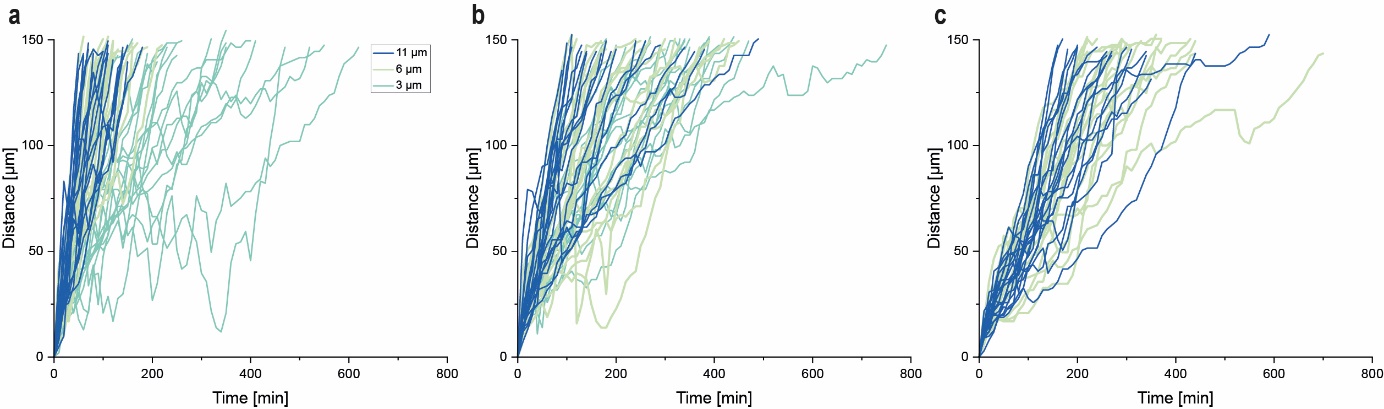


Supplementary Fig. 1: Kymographs of MDA-MB-231 cells in different conditions. a) Kymographs in 3 µm, 6 µm, and 11 µm channels at 0 mV mm^-1^. b) Kymographs in 3 µm, 6 µm, and 11 µm channels at 500 mV mm^-1^. c) Kymographs in 3 µm, 6 µm, and 11 µm channels at 1000 mV mm^-1^.


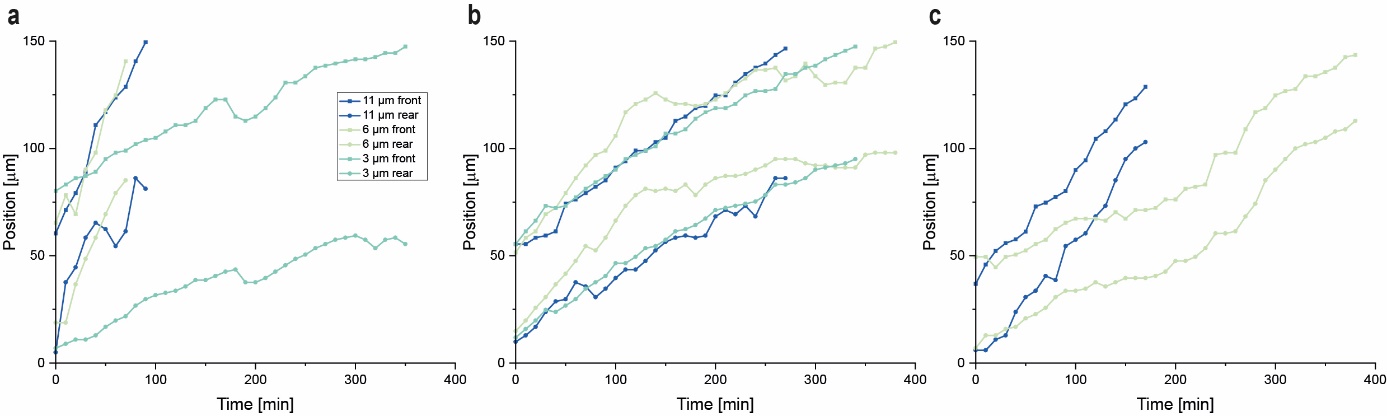


Supplementary Fig. 2: Exemplary front and rear trajectories of MDA-MB-231 cells inside the different channel dimensions. a) Trajectories on an individual cell inside 3 µm, 6 µm, and 11 µm channels at 0 mV mm^-1^. b) Trajectories on an individual cell inside 3 µm, 6 µm, and 11 µm channels at 500 mV mm^-1^. c) Trajectories on an individual cell inside 3 µm, 6 µm, and 11 µm channels at 1000 mV mm^-1^.


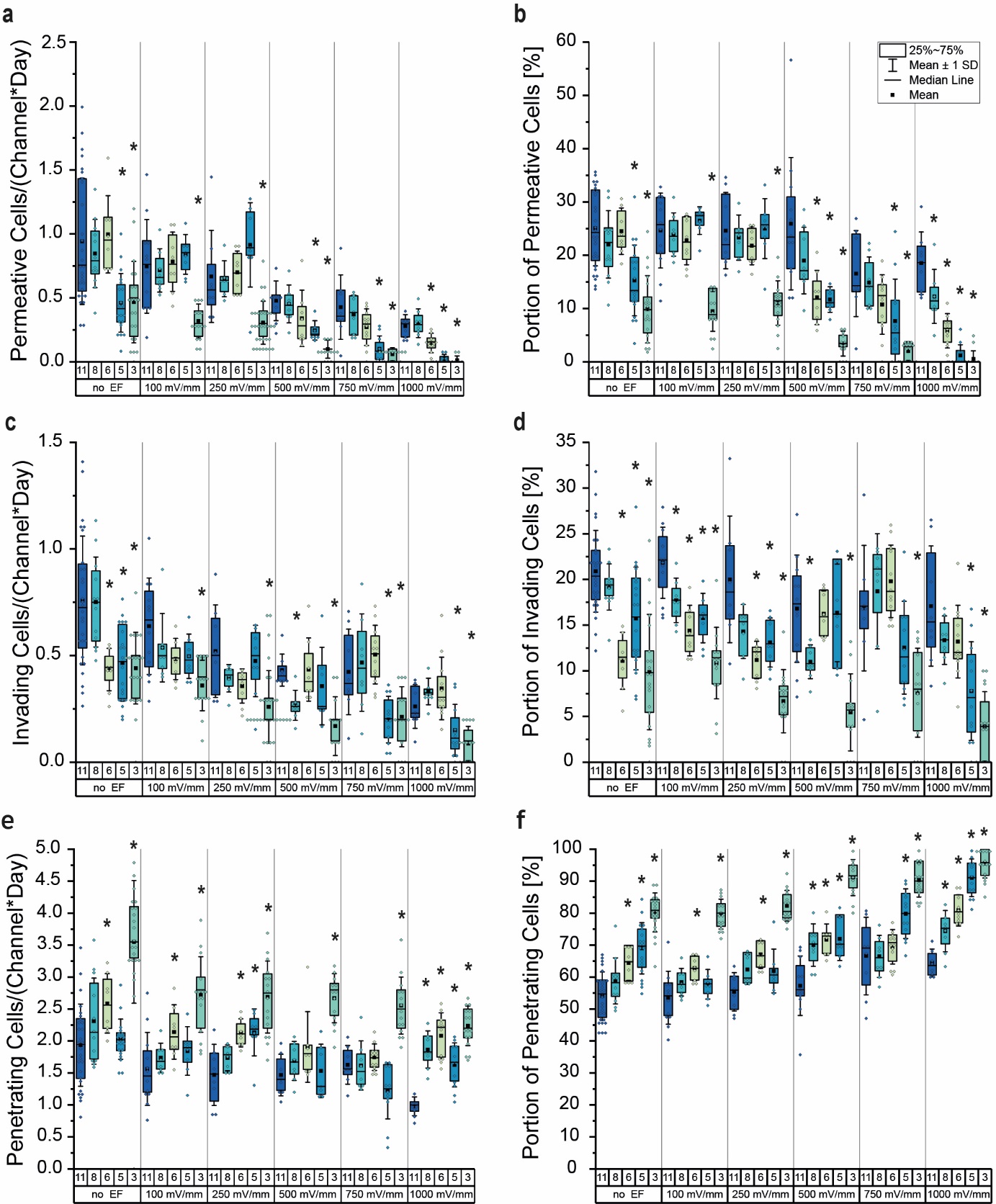


Supplementary Fig. 3: Graphs on all channel interactions of MDA-MB-231. a) Entire quantification of permeating cells per channel over one day (24 h) at different electrical fields. b) Entire proportion of permeating cells in different channel sizes at different electrical fields. c) Entire quantification of invading cells per channel over one day (24 h) at different electrical fields. d) Entire proportion of permeating cells in different channel sizes at different EFs. e) Entire quantification of penetrating cells per channel over one day (24 h) at different electrical fields. f) Entire proportion of permeating cells in different channel sizes at different electrical fields (n_image sequence_ ≥ 8; * p < 0.05 in comparison to 11 μm-confinement in according field strength, Mann-Whitney-Test).


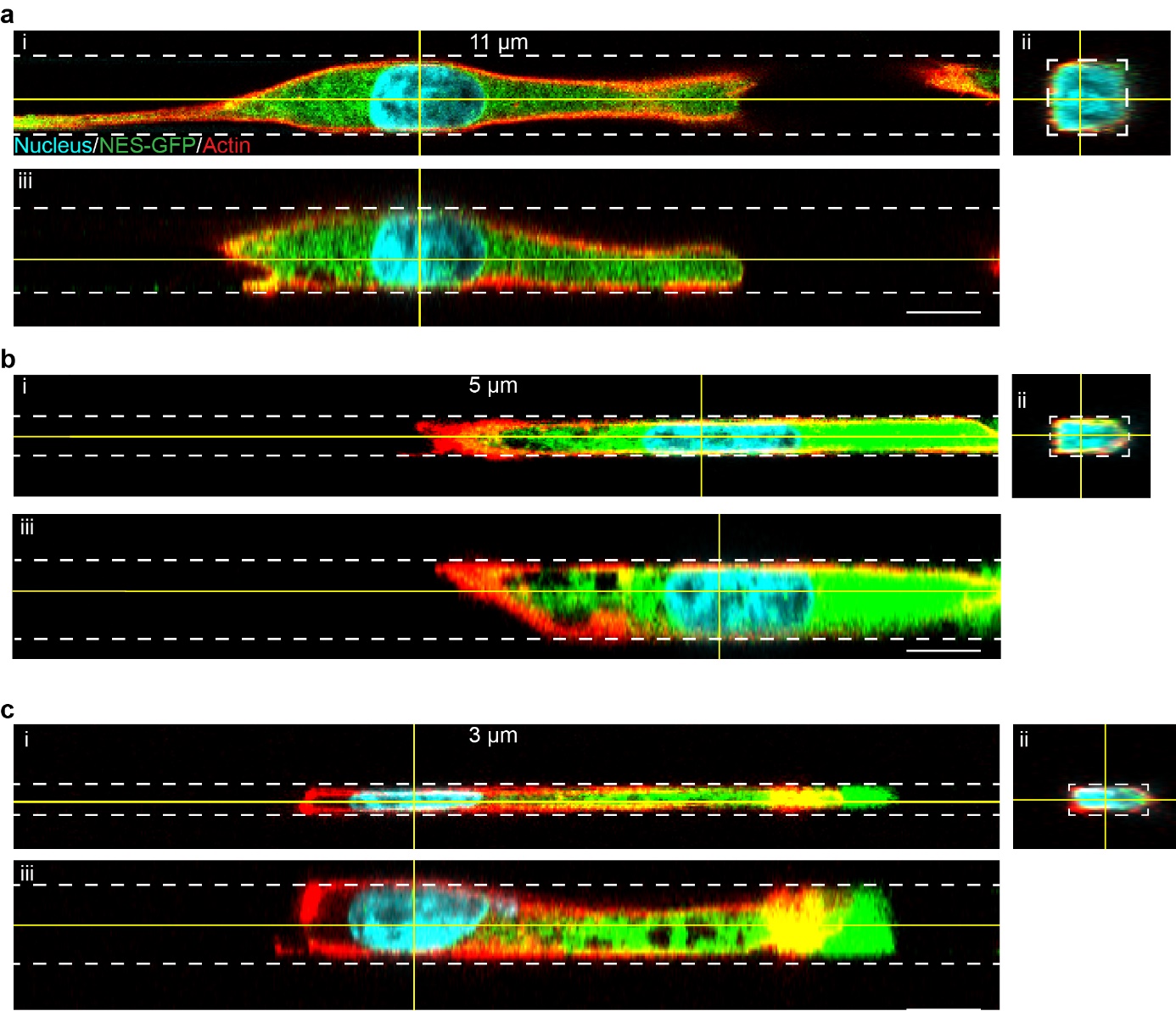


Supplementary Fig. 4: Exemplary fluorescent images of MDA-MB-231 cells inside the microchannels. a) Merged image in 11 µm confinement. b) Merged image in 5 µm confinement. c) Merged image in 3 µm confinement. i) Top (XZ) view of the cell. ii) Front (YZ) view of the cells. ii) Side (XY) view of the cell. (White dashed lines resemble the borders of the microchannel; scale bar 10 µm.)

Supplementary Video 1: Exemplary Movie of MDA-MB-231 cells migrating through 3 µm confinements over 24 h.

Supplementary Video 2: Exemplary Movie of MDA-MB-231 cells migrating through 11 µm confinements over 24 h.
